# Oxidative stress triggers aggregation of GFP-tagged Hsp31p, the budding yeast environmental stress response chaperone, and glyoxalase III

**DOI:** 10.1007/s12192-017-0868-8

**Published:** 2017-12-20

**Authors:** Urszula Natkańska, Adrianna Skoneczna, Marek Skoneczny

**Affiliations:** 1 Institute of Biochemistry and Biophysics, Department of Genetics, Polish Academy of Sciences, Pawińskiego 5A, 02-106 Warszawa, Poland; 2 Institute of Biochemistry and Biophysics, Laboratory of Mutagenesis and DNA Repair, Polish Academy of Sciences, Pawińskiego 5A, 02-106 Warszawa, Poland

**Keywords:** *Saccharomyces cerevisiae*, Environmental stresses, Protein stability, Protein aggregates

## Abstract

The *Saccharomyces cerevisiae* Hsp31p protein belongs to the ubiquitous DJ-1/ThiJ/PfpI family. The most prominent member of this family is human DJ-1; defects of this protein are associated with Parkinson’s disease pathogenesis. Numerous recent findings reported by our group and others have revealed the importance of Hsp31p for survival in the post-diauxic phase of cell growth and under diverse environmental stresses. Hsp31p was shown to possess glutathione-independent glyoxalase III activity and to function as a protein chaperone, suggesting that it has multiple cellular roles. Our previous work also revealed that *HSP31* gene expression was controlled by multiple stress-related transcription factors, which mediated *HSP31* promoter responses to oxidative, osmotic, and thermal stresses, toxic products of glycolysis, and the diauxic shift. Nevertheless, the exact role of Hsp31p within budding yeast cells remains elusive. Here, we aimed to obtain insights into the function of Hsp31p based on its intracellular localization. We have demonstrated that the Hsp31p-GFP fusion protein is localized to the cytosol under most environmental conditions and that it becomes particulate in response to oxidative stress. However, the particles do not colocalize with other granular subcellular structures present in budding yeast cells. The observed particulate localization does not seem to be important for Hsp31p functionality. Instead, it is likely the result of oxidative damage, as the particle abundance increases when Hsp31p is nonfunctional, when the cellular oxidative stress response is affected, or when cellular maintenance systems that optimize the state of the proteome are compromised.

## Introduction

*Saccharomyces cerevisiae* Hsp31p belongs to the ubiquitous DJ-1/ThiJ/PfpI superfamily, which is mainly composed of poorly characterized proteins present in almost all prokaryotes and eukaryotes sequenced to date. The most prominent of the better-known family members is DJ-1, which plays important roles in protecting humans against cancer, cardiovascular disease, and neurodegeneration symptoms (Chan and Chan 2015). Unsurprisingly, determining the roles of DJ-1 and its orthologues from other organisms has become a priority in aging Western societies. Crystal structures of these proteins have been available for many years (Wilson et al. 2003; Wilson et al. 2004; Wilson et al. 2005; Seo et al. 2012), and recently, a large amount of data have revealed their molecular functions. DJ-1 is by far the most frequently studied protein in this family. Nevertheless, the exact roles of DJ-1 in human cells (Chan and Chan 2015), *Candida albicans* Glx3, which belongs to the same family (Hasim et al. 2014), and *S. cerevisiae* Hsp31p (Natkańska et al. 2017; Aslam and Hazbun 2016) are not fully understood. One helpful clue for deciphering their biological role(s) is their cellular localization. DJ-1 is apparently localized to multiple compartments. The first evidence of the mitochondrial, cytosolic, and nuclear localization of DJ-1 was presented more than a decade ago (Bonifati et al. 2003). The presence of this protein in the mitochondria has been shown to depend on the protein’s functionality and oxidation state at the conserved Cys106 (Canet-Avilés et al. 2004; Zhang et al. 2005). This early finding has since been confirmed, and numerous other studies have expanded our knowledge; however, the mechanism by which DJ-1 enters mitochondria has remained mysterious to date (Kojima et al. 2016). The nuclear localization of DJ-1 has been implicated in oxidative stress signaling (Kim et al. 2012; Björkblom et al. 2014). More recently, DJ-1 has been shown to be associated with lipid rafts on the cell membrane (Kim et al. 2013) and with the Golgi apparatus (Usami et al. 2011). The presence of this protein in multiple locations within the cell suggests that it has multiple functions (“moonlighting” protein), which is conceivable for a protein involved in protection against oxidative damage.

Data regarding the localization of DJ-1/ThiJ/PfpI superfamily members from other species are scarce. The same is also true for *S. cerevisiae* Hsp31p, and most available data have been derived from whole-proteome localization studies (Huh et al. 2003; Tkach et al. 2012). Those studies were enabled by the availability of a collection of strains bearing genomic constructs encoding C-terminal green fluorescent protein (GFP) fusions of most budding yeast cellular proteins. Their results showed that Hsp31p-GFP was localized to the cytosol. These localization data were mainly collected under standard growth conditions; however, Hsp31p exhibits responses to multiple environmental stresses. Numerous stress response proteins are present in specific cellular subcompartments, and some change their localization patterns following stress exposure (Tkach et al. 2012; Breker et al. 2013). Conversely, some types of stress are often associated with certain cellular regions. For instance, peroxisomes and mitochondria are the sources of oxidative stress (Antonenkov et al. 2010; Bleier and Dröse 2013). Genotoxic stress is distinctive to DNA, which is present in the nucleus and in organelles such as mitochondria and chloroplasts (Skoneczna et al. 2015). The unfolded protein response is characteristic of the endoplasmic reticulum (Wu et al. 2014), whereas proteins that provide protection against heat shock are mainly localized to the cytoplasm (Verghese et al. 2012). Various stress signaling proteins migrate to and from the nucleus depending on the stress conditions (Plotnikov et al. 2011). In addition to lipid bilayer-demarcated organelles, many proteinaceous structures have been found to be constantly present or to appear following stress exposure, such as stress granules, P-bodies (Olszewska et al. 2012), the intranuclear quality control compartment (INQ), the insoluble protein deposit compartment (IPOD), and cytosolic stress-induced aggregates (CytoQ) (Miller et al. 2015). In addition to these purposeful assemblies, eukaryotic cells can be cluttered with protein aggregates, which are generally regarded as pathogenic or at least as a burden that reduces cellular fitness. The best-known examples in humans are aggregates that form in the central nervous system and lead to neurodegenerative diseases, including Alzheimer’s, Parkinson’s, Huntington’s, Creutzfeldt-Jacob, tauopathies and amyotrophic lateral sclerosis (see recent reviews (Renner and Melki 2014; Sami et al. 2017)). The latter disease may result from the inefficient disposal of stress granules (Monahan et al. 2016). The yeast *S. cerevisiae* is also susceptible to protein aggregation-induced pathologies, and prion development and propagation have been extensively studied using this model organism (Wickner et al. 2015). Consequently, budding yeast is also a convenient model to study the molecular mechanisms of human neurodegenerative diseases (Fruhmann et al. 2016). As mentioned above, whole-proteome localization studies have not provided any helpful information regarding the subcellular localization of Hsp31p. However, we did not find this result discouraging, especially because most of the stresses that Hsp31p seems to protect against were not represented in those screens.

In the present study, we analyzed the subcellular localization of Hsp31p under various stress conditions to resolve this outstanding question and bring us closer to a complete understanding of its intracellular role. The results of this analysis confirmed earlier whole-proteomic findings that the Hsp31p-GFP fusion protein is localized to the cytosol under standard growth conditions. Exposure of yeast cells to some of the stresses that induce *HSP31* gene expression led to increased protein levels, with no change in its subcellular localization. Interestingly, we also found that in addition to its uniform cytosolic distribution, Hsp31p-GFP assembled into foci within the cytosol under oxidative stress conditions and in post-diauxic cells. These foci did not colocalize with markers of known particulate structures present in budding yeast cells, and their average numbers increased in cells lacking proteins involved in protein disaggregation. These foci were also more abundant in the strain expressing Hsp31p-GFP lacking Cys138, which is crucial for Hsp31p enzymatic function, or in the strain with a defective oxidative stress response. Our data strongly suggest that these particles are aggregates with no functional assignment.

## Materials and methods

### Yeast strains and plasmids

The *S. cerevisiae* strains used in this study are listed in Table 1, and the plasmids used are listed in Table 2. Single-copy plasmids encoding various GFP-tagged Hsp31p proteins were constructed in several steps. To construct the single-copy YCpHSP31C➔A plasmid, the *Eco*RI-*Nae*I fragment containing a full-length mutated *HSP31* gene (encoding a protein with a Cys138-to-Ala substitution), together with its promoter (up to −453 bp) and terminator (down to +277 bp), was excised from pRSHSPC➔A (Natkańska et al. 2017) and cloned into a YCp50 centromeric vector (Rose et al. 1987), linearized with the *Eco*RI and *Nru*I enzymes. To construct the YCpHSP31 plasmid containing the native *HSP31* gene, the *Hind*III-*Sac*I insert was excised from pRSHSP31 (Natkańska et al. 2017) and cloned into the YCpHSP31C➔A plasmid, linearized with the same enzymes.

**Table 1:** Yeast strains used in this study

Strains	Genotype	Source
BY4741	*MATa his3Δ1 leu2Δ0 met15Δ0 ura3Δ0*	Open Biosystems^a^
*yap1∆*	*MATa his3Δ1 leu2Δ0 met15Δ0 ura3Δ0 yap1::KanMX*	Open Biosystems^a^
*btn2∆*	*MATa his3Δ1 leu2Δ0 met15Δ0 ura3Δ0 btn2::KanMX*	Open Biosystems^a^
*hsp104∆*	*MATa his3Δ1 leu2Δ0 met15Δ0 ura3Δ0 hsp104::KanMX*	Open Biosystems^a^
*hsp42∆*	*MATa his3Δ1 leu2Δ0 met15Δ0 ura3Δ0 hsp42::KanMX*	Open Biosystems^a^
*cue5∆*	*MATa his3Δ1 leu2Δ0 met15Δ0 ura3Δ0 cue5::KanMX*	Open Biosystems^a^
*ssa2∆*	*MATa his3Δ1 leu2Δ0 met15Δ0 ura3Δ0 ssa2::KanMX*	This study
BY HSP31GFP	*MATa his3Δ1 leu2Δ0 met15Δ0 ura3Δ0 HSP31GFP::HIS3MX6*	Huh et al. (2003)
BY WT Hsp42RFP	as BY4741+ HSP42-yTagRFP-T	Malcova et al. (2016)
BY WT Hsp104RFP	as BY4741+ HSP104-yTagRFP-T	Malcova et al. (2016)
1-5V-H19 [PSI^−^, PIN^−^]	*MAT* ***a*** *ade2-1 SUQ5 can1-100 leu2-3112 ura3-52 [psi* ^*−*^ *]*	Ter-Avanesyan et al. (1994)
1-5V-H19 [PSI^−^, PIN^+^]	*MAT* ***a*** *ade2-1 SUQ5 SUP35-C leu2-3112 ura3-52 can-100 [psi* ^*−*^ *] [PIN* ^*+*^ *]*	Ter-Avanesyan et al. (1994)
74D-694 [PSI^−^]	*MAT* ***a*** *ade1–14(UGA) trp1–289(UAG) his3-Δ200 leu2–3112 ura3–52*	Chernoff et al. (1995)
74D-694 [PSI^+^]	*MAT* ***a*** *ade1–14(UGA) trp1–289(UAG) his3-Δ200 leu2–3112 ura3–52* (Sup35 overproduction)	Chernoff et al. (1995)

^a^All deletion strains were obtained from the *Saccharomyces* Genome Deletion Project (http://wwwsequence.stanford.edu/group/yeast_deletion_project/) through Open Biosystems (Huntsville, AL, USA). The *ssa2∆* strain was newly constructed in the BY4741 background using the *KanMX* deletion cassette and PCR-amplified with genomic DNA from the homozygote *ssa2∆*/*ssa2∆* from the same collection as the template using the ssa2up and ssa2lw primers (see Table 3). The resulting clone was confirmed by PCR using the ssa2lw2 and Kan_up primers

**Table 2:** Plasmids used in this study

Plasmids	Source
YCpHSP31	This study
YCpHSP31C-A	This study
pUG35HSP31	This stud
pUG36promHSP31	This study
pUG36HSP31	This study
YCpHSP31GFP	This study
YCpHSP31GFP C-A	This study
YCpGFPHSP31	This study
YCpGFPHSP31 C-A	This study
YEpHSP31	Natkańska et al. (2017)
YEpHSP31C-A	Natkańska et al. (2017)
pJH208	Hu et al. (2008)
pFLHSP104	Chacinska et al. (2001)
pRS316-mRFP-SKL	Fagarasanu et al. (2009)
Sec7mRFP	Matsuura-Tokita et al. (2006)
pRP1574Edc3RFP	Buchan et al. (2008)
pRP1574Dcp2mCherry	Buchan et al. (2008)

**Table 3:** Primers used in this study

Primer	Primer sequence (5′ to 3′)
Hspcup	GTCTAGAAACCATTATTATCATGA
HspSalp	TTTGTCGACGTTTTTTAAAGCGTCGATGGA
pug36-1	TAGAGCTCAAGCCCATAAAAAAATCACG
pug36-2	GCACTATTATAAATTTGTTTGAGTTTTATCTGTGT
pug36-3	TATCTAGAGCCCCAAAAAAAGTTTTACTC
pug36-4	ATCCCGGGGATAAATGCTGTGCAAC
Ssa2up	ATTCGTAGTATTTCCGAAGCT
Ssa2lw	ATGACCGATATTCCTAGTCT
ssa2lw2	CACCAGCTACCACCACTT
Kan_up	TGATTTTGATGACGAGCGTAAT

To construct the plasmid encoding the C-terminal fusion of Hsp31p with GFP, the whole *HSP31* open-reading frame (ORF) with its promoter (upstream to −453 bp) was PCR-amplified using the Hspcup and HspSalp primer pair and the YCpHSP31 plasmid as a template. The PCR product was digested with *Xba*I and *Sal*I and ligated into the pUG35 (Güldener and Hegemann, unpublished data, GenBank: AF298787.1) vector, linearized with the same restriction enzymes. The insert of the resulting pUG35HSP31 plasmid was sequenced to confirm the absence of point mutations. Then, the plasmid was digested with *Mlu*I, blunt-ended with Klenow, and digested with *Xba*I. The *Mlu*I-*Xba*I fragment containing the *HSP31* C-terminal GFP fusion was ligated to the larger fragment of the YCpHSP31 plasmid digested with *Pvu*II and *Xba*I to obtain the YCpHSP31GFP plasmid. To construct the YCpHSP31GFPC➔A plasmid encoding C-terminal Hsp31p-GFP with the Cys138➔Ala modification, the 1796-bp *Vsp*1-*Age*I fragment of the YCpHSP31GFP plasmid was replaced with a fragment of the same size, excised from the YCpHSP31C➔A plasmid by partial digestion with *Age*I and complete digestion with *Vsp*I.

To construct the plasmids encoding N-terminally GFP-tagged Hsp31p, the *HSP31* promoter (up to −453 bp) was PCR-amplified with the primer pair pug36-1 and pug36-2 using the YCp50HSP31 plasmid as a template. The resulting product was digested with *Spe*I and *Sac*I and ligated into the pUG36 (Güldener and Hegemann, unpublished data, GenBank: AF298791.1) vector, linearized with the *Sac*I and *Xba*I restriction enzymes to create the pUG36promHSP31 plasmid, in which the *HSP31* promoter replaced the *MET25* promoter upstream of the GFP-encoding sequence. This plasmid was digested completely with *Sac*I and partially with *Xba*I to enable insertion of the DNA fragment containing the whole *HSP31* ORF and its 3′ untranslated region (UTR) (down to +277 bp), which were obtained by PCR amplification with the primer pair pug36-3 and pug36-4 using YCp50HSP31 as a template, followed by digestion with *Xma*I and *Xba*I. The insert of the resulting pUG36HSP31 plasmid was sequenced to confirm the absence of point mutations. Finally, to obtain the YCp50GFPHSP31 plasmid encoding the N-terminal GFP-Hsp31p fusion under control of the *HSP31* promoter, the *Xba*I-*Sac*I fragment from pUG36HSP31 was inserted into the same sites of the YCp50HSP31 vector. To construct the sister plasmid YCp50GFPHSP31C➔A encoding the N-terminal GFP-Hsp31p fusion (but with Cys138 replaced with Ala), the *Ecl*136II-*Eco*RI fragment was excised from YCp50HSP31C➔A and inserted into YCp50GFPHSP31, linearized with the *Zra*I and *Eco*RI enzymes. The resulting intermediate construct was cut with *Bsu*36I and self-ligated. The plasmids were maintained and propagated in bacteria using standard procedures and introduced into yeast cells using the lithium acetate method.

### Growth conditions

Yeast cells were grown at 28 °C in either rich yeast extract-peptone-dextrose (YPD) medium (1% yeast extract, 2% peptone, and 2% glucose) or synthetic complete (SC) medium (0.67% yeast nitrogen base and 2% glucose) supplemented with drop-out amino acid mixtures to maintain the selective pressure. To create solid media, 2% agar was added.

### Fluorescence microscopy

Yeast strains grown overnight in selective SC medium lacking uracil were inoculated into fresh SC medium and grown to mid-log phase. The cultures were aliquoted, and various stressors were added at the concentrations indicated in the figure legends. Then, the cultures were grown at 28 °C for an additional 2 h. Post-diauxic-phase cells were obtained by growth for 24 h in SC medium lacking uracil. Prior to microscopic examination, the cells were washed twice with 1× PBS and resuspended in the same buffer. The cells were imaged on a Zeiss Axio Imager.M2 fluorescence microscope with a 100× objective and 38HE-GFP or 20HE-rhodamine filter sets. For quantitative analysis, the percentages of cells with fluorescent foci were scored by examining 600 cells in at least three independent experiments. To assure reproducibility between experiments, all images were collected with the same microscope settings. Statistical significance was determined using Student’s *t* test.

### Redox Western

Redox Western blot analysis of Hsp31p was performed with cytosolic rxYFP (a redox-sensitive protein) as the control according to a previously described method (Østergaard et al. 2004). Briefly, the BY *hsp31*∆ strain was transformed with the pJH208 plasmid encoding the redox-sensitive rxYFP control protein (Hu et al. 2008) and either the YEpHSP31 or YEpHSPC➔A plasmid (Natkańska et al. 2017) encoding native Hsp31p or Hsp31p lacking Cys138, respectively. The cells were grown overnight in selective SC medium without uracil and leucine. The cultures were inoculated into YPD medium and grown to a density of 1–2 × 10^7^ cells ml^−1^. Then, the cultures were treated with 0.1 mM cumene-OOH to induce oxidative stress and grown at 28 °C for an additional 2 h. The yeast cells were transferred to fresh YPD medium supplemented with 100 mM 4,4′-dithiodipyridine (4-DPS) (Sigma), 50 mM dithiothreitol (DTT) (Thermo Scientific) or no supplementation and incubated at 28 °C for an additional 20 min. The cell cultures were acid-quenched with trichloroacetic acid (Sigma) (15% (*w*/*v*) final concentration) at 4 °C for 20 min. Then, the cells were harvested by centrifugation and resuspended in 250 μl of 10% trichloroacetic acid. Next, glass beads were added, and the cells were homogenized by vortexing five times for 1 min per round and kept on ice between rounds. The cells were pelleted by centrifugation, and the cell debris was resuspended in 125 μl of 1× non-denaturing SDS sample buffer (w/o DTT) containing 40 mm *N*-ethylmaleimide (Sigma). After a 10-min incubation at room temperature, the proteins were separated on a 16% Tris-glycine gel. Reduced and oxidized forms of the proteins were analyzed by Western blotting using an anti-GFP horseradish peroxidase (HRP)-conjugated antibody (Vector Laboratories) for rxYFP and an anti-Hsp31p HRP-conjugated antibody for Hsp31p (Everest Biotech Ltd). Actin (the loading control) was detected with mouse monoclonal anti-actin primary antibody (Millipore) and goat anti-mouse-HRP (Dako) secondary antibody.

## Results

### During oxidative stress, GFP-tagged Hsp31p localization partially changes from the cytosol to unspecified particles within the cytosol

**Fig. 1:**
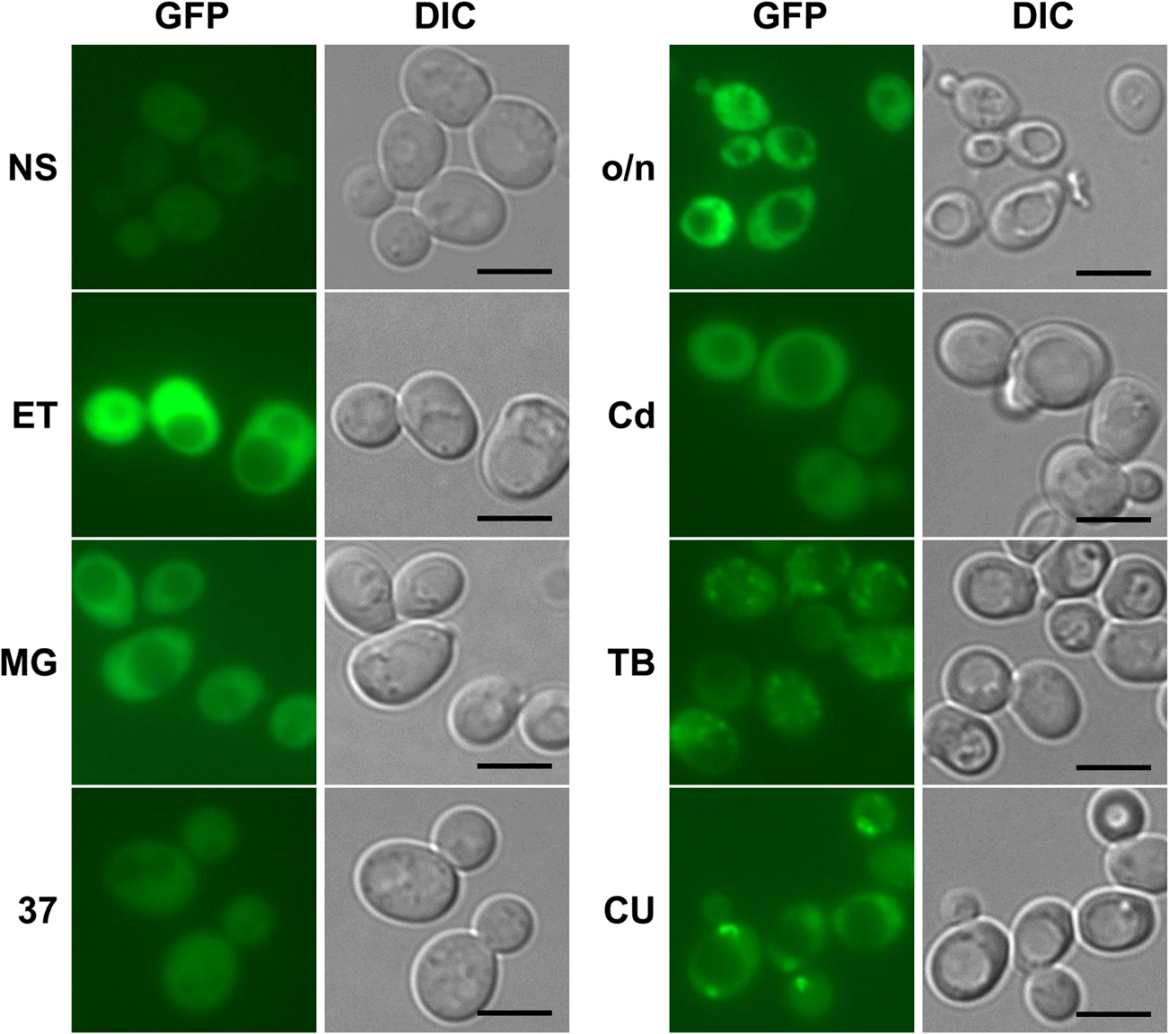
Intracellular localization of C-terminally GFP-tagged Hsp31p expressed from the genomic locus under standard growth conditions and after exposure to various stresses. *S. cerevisiae* cells of the YDR533C clone from the YeastGFP collection (Huh et al. 2003) were grown at 28 °C in SC medium either overnight to reach the post-diauxic growth phase (o/n) or to mid-exponential phase (1–2 × 10^7^ cells × ml^−1^) (NS), followed by 2 h of exposure to 7% ethanol (ET), 0.5 mM methylglyoxal (MG), 1.25 μM cadmium chloride (Cd), 0.2 mM tert-butyl-OOH (TB), or 0.1 mM cumene-OOH (CU). To apply heat-stress conditions, yeast cells pregrown to mid-exponential phase at 23 °C were transferred to a 37 °C water bath and incubated for 30 min with shaking. Following incubation under stress conditions, the cells were spun down and resuspended in PBS, and GFP fluorescence was examined on an Axio Imager.M2 fluorescence microscope using a 100× objective and 38HE-GFP filter set (Zeiss). Nomarski optics were used for bright-field cell visualization (DIC). Scale bar: 5 μm

**Fig. 2:**
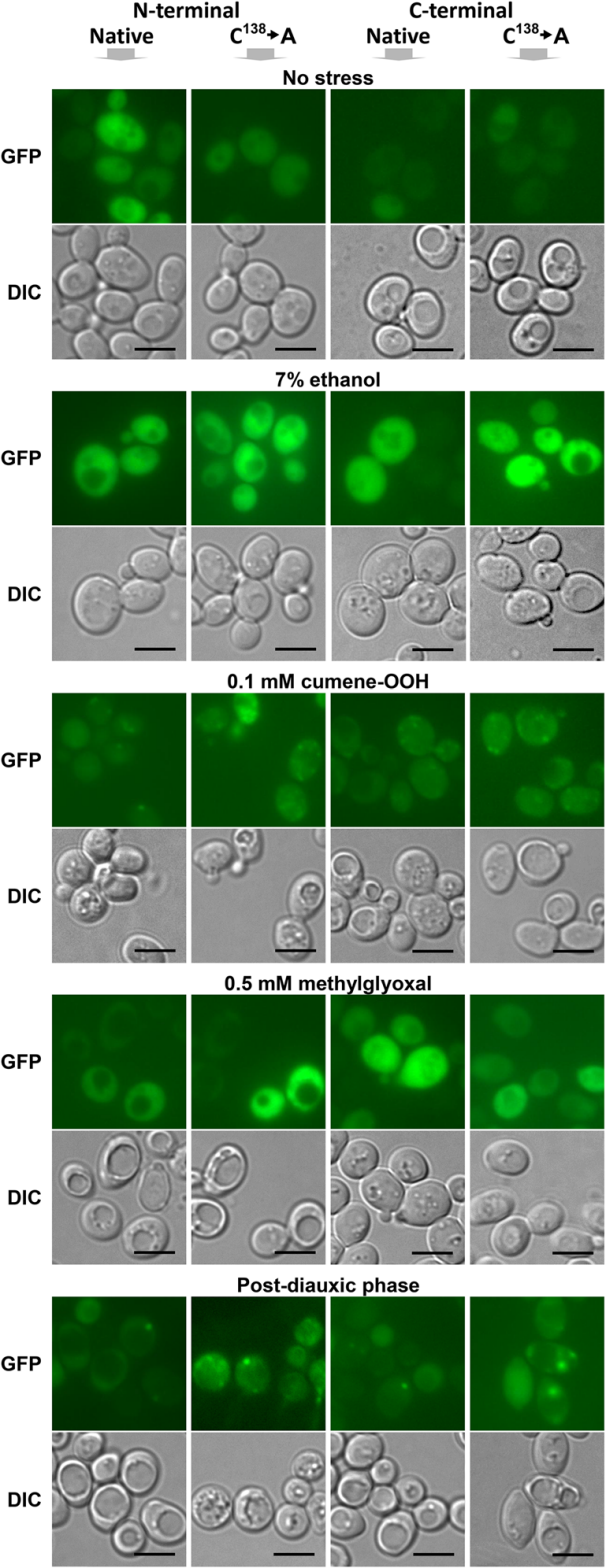
Neither the presence of the Cys138 amino acid residue nor the placement of the GFP tag is critical for the intracellular distribution of GFP-tagged Hsp31p. Wild-type BY4741 cells were transformed with single-copy YCp50-derivative plasmids bearing constructs encoding either native Hsp31p or Hsp31p lacking the conserved cysteine residue (C^138^➔A), which were each tagged with GFP at the N- or C-terminus (see the “Materials and methods” section). Transformed cells were grown, exposed to stressors, and visualized by fluorescence microscopy as described in Fig. 1 legend. Scale bar: 5 μm

**Fig. 3:**
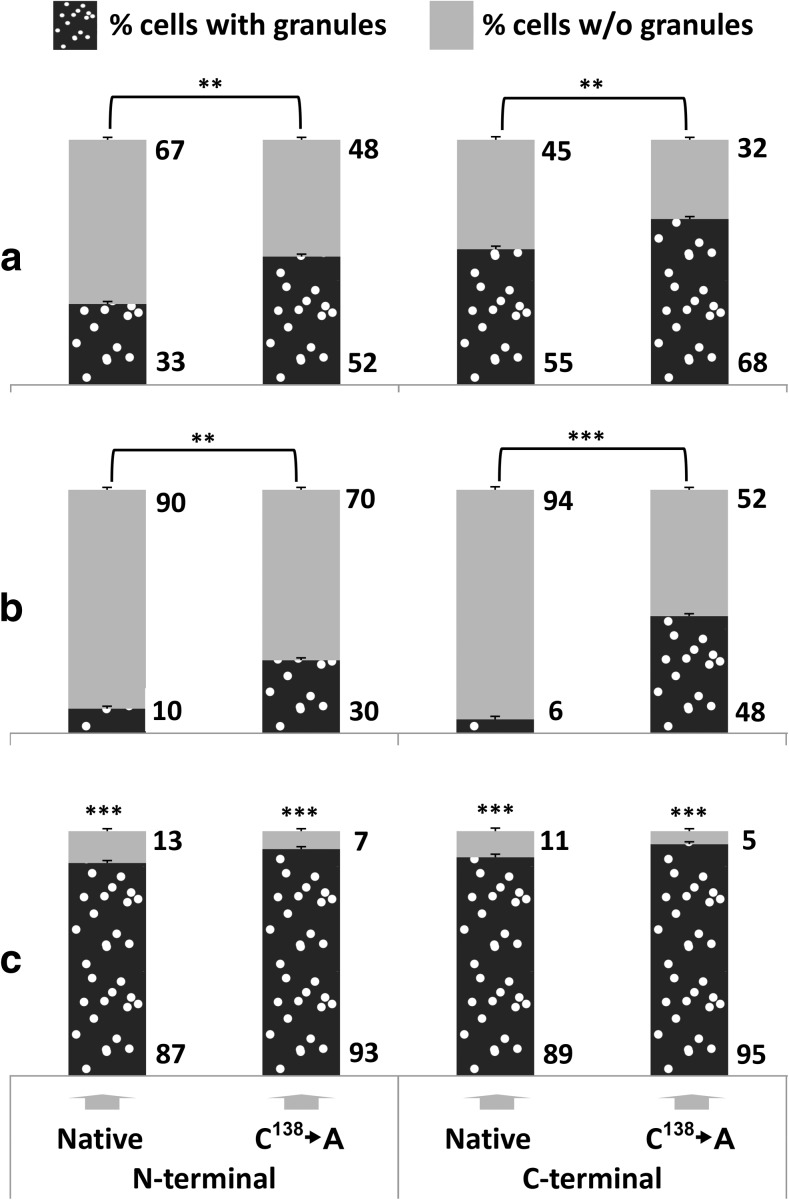
Quantitative analysis of the intracellular distribution of GFP-tagged Hsp31p in wild-type and *yap1Δ* strains exposed to oxidative stress and in wild-type post-diauxic cells. Wild-type (**a**, **b**) and *yap1Δ* (**c**) cells were transformed with the same plasmids described in Fig. 2 legend. Wild-type (**a**) and *yap1Δ* (**c**) transformant cells were grown to mid-exponential phase (1–2 × 10^7^ cells × ml^−1^) in SC medium and exposed to 0.1 mM cumene-OOH for 2 h. Wild-type transformant cells (**b**) were grown overnight in SC medium. Then, the cells were visualized by fluorescence microscopy as described in Fig. 1 legend. Cells of the transformant strains were scored for the presence of green fluorescent particles. Each data point is the average of three biological replicates, with 200 cells counted per replicate; the standard deviations are represented by error bars. Statistical significance was calculated with Student’s *t* test: *** *p* < 0.001, ** 0.001 < *p* < 0.05. Panels (**a**) and (**b**) show the significance of the differences between the native proteins and proteins lacking the Cys138 residue. The significance shown in panel (**c**) refers to the differences between the data obtained for the *yap1Δ* deletion strain and the wild type (**a**) transformed with the respective plasmids

**Fig. 4:**
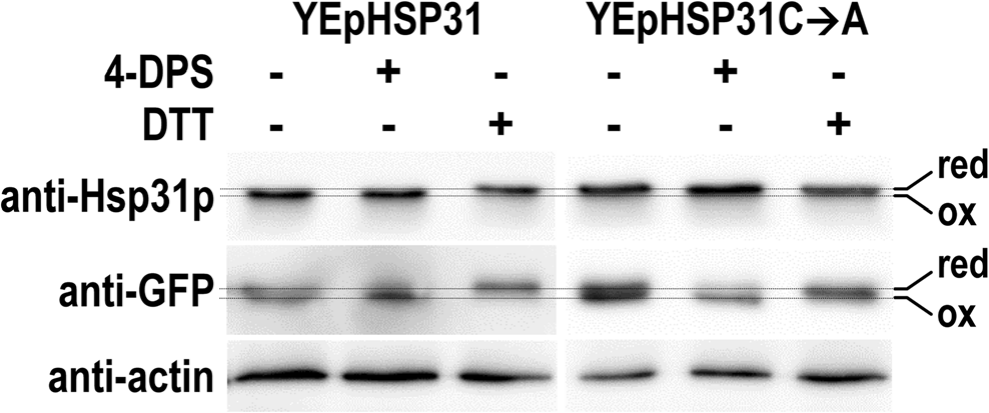
The conserved Cys138 amino acid residue of Hsp31p is susceptible to oxidation in cells exposed to oxidative stress. *hsp31Δ* strains transformed with multi-copy plasmids expressing either native Hsp31p (YEpHSP31) or Hsp31p without the Cys138 residue (YEpHSP31C➔A) and with the plasmid expressing the reference redox-sensitive rxYFP protein were grown to exponential phase, exposed to oxidative stress and then treated with either oxidizing (4-DPS) or reducing (DTT) agents or left untreated. The electrophoretic mobility of Hsp31p and rxYFP was analyzed by non-denaturing SDS polyacrylamide gel electrophoresis, followed by Western blotting and immunodetection of proteins with the anti-Hsp31p-HRP, anti-GFP-HRP primary conjugated antibodies, or anti-actin antibodies as the loading control (see the “Materials and methods” section for more details). The “red” and “ox” terms denote the reduced and oxidized forms of the proteins, respectively

The collection of strains bearing C-terminal genomic fusions of most *S. cerevisiae* ORFs with the sequence encoding GFP (Huh et al. 2003) represents a very convenient tool that is highly praised by the yeast research community. The whole collection or individual strains have been utilized in countless studies. According to the Web of Science (http://www.webofknowledge.com/), the article describing the creation of this collection and its use to localize *S. cerevisiae* proteins has been cited 2492 times as of November 2017. Therefore, in our attempt to determine the localization of Hsp31p within budding yeast cells, we started by simply examining existing collections of images supplementing whole-genomic studies, including the original study in which cells were grown to mid-log phase in standard SD medium (Huh et al. 2003) and more recent studies examining localization changes following genotoxic stress (Tkach et al. 2012), unfolded protein stress, oxidative stress, and starvation (Breker et al. 2013). Under all these conditions, Hsp31p-GFP was uniformly distributed in the cytosol. This subcellular localization pattern is common to hundreds of *S. cerevisiae* proteins but does not provide any useful information regarding the intracellular role of Hsp31p. Nevertheless, we have previously demonstrated that Hsp31p is a multi-stress response protein (Natkańska et al. 2017; Skoneczna et al. 2007) that is responsive to the diauxic shift and to oxidative, osmotic, and thermal stresses. Moreover, strongest expression of the *HSP31* gene was observed following exposure of yeast cells to toxic products of fermentation (ethanol and methylglyoxal). Because these stressors were not used in any global localization screens with the yeast GFP protein fusion collection, we initially tested whether Hsp31p-GFP localization in cells of the collection clone changed upon exposure to the same environmental stresses employed in our recent study (Natkańska et al. 2017). The results of this experiment, which are presented in Fig. 1, demonstrated that the cytosolic localization in response to ethanol, methylglyoxal, and thermal stresses was indistinguishable from that under standard growth conditions, except that Hsp31p-GFP fluorescence was much stronger in the presence of glycolysis products. However, under oxidative stress, and to some extent after the diauxic shift, the fluorescence was observed in particles distributed within the cytosol. We investigated whether the subcellular distribution of Hsp31p was influenced by the location of the GFP tag relative to the Hsp31p polypeptide. Because our group (Natkańska et al. 2017) and others (Bankapalli et al. 2015) have recently demonstrated the importance of the conserved Cys138 residue for Hsp31p functionality, we assessed whether the presence of this residue influenced the subcellular localization of Hsp31p, knowing that the side-chain of the cysteine residue might be prone to oxidation under oxidative-stress conditions, leading to inactivation of the enzymatic activity of Hsp31p. To test this possibility, we constructed single-copy YCp50-based plasmids expressing both C-terminal and N-terminal fusions of Hsp31p with GFP under the native *HSP31* promoter that either contained or lacked the Cys138 residue. The plasmids were introduced into the haploid BY4741 strain. As documented in Fig. 2, the placement of the GFP tag did not seem to influence the localization of the fusion protein in unstressed or stressed cells. Moreover, the absence of the cysteine residue did not prevent particle formation under oxidative stress or in post-diauxic cells. In contrast, quantitative analysis revealed that the particles appeared significantly more frequently (see Fig. 3a, b). We also examined whether dysfunction of the cellular response to oxidative stress manifested as a change in Hsp31p-GFP localization. As shown in Fig. 3c, following exposure of the *yap1Δ* strain (BY4741 genetic background), which was devoid of the oxidative-stress response regulator Yap1p, to oxidative stress conditions, the Hsp31p-GFP particles were much more abundant and were present in almost every cell. This phenomenon may result from synergy between the exogenous stress caused by the addition of cumene hydroperoxide and the inability of *yap1Δ* cells to properly react to this stress. Nevertheless, no Hsp31p-GFP particles were detected in unstressed *yap1Δ* cells, similar to unstressed wild-type cells (data not shown). To obtain a more complete picture of the effects of oxidative stress on Hsp31p, we also assessed whether the Cys138 residue of this protein was oxidized under these conditions. For this purpose, we employed yeast cells expressing either native Hsp31p or Hsp31p lacking Cys138, as well as the diagnostic rxYFP protein, which exhibits an altered migration speed depending on its redox state by non-reducing SDS polyacrylamide gel electrophoresis. Cells were subjected to oxidative stress conditions for 2 h and divided into three groups as follows: no further treatment or incubation under either reducing (50 mM dithiothreitol) or oxidizing (100 mM 4,4′-dithiodipyridine) conditions. Protein migration shifts were detected by electrophoresis followed by Western blotting with an anti-GFP antibody for rxYFP and an anti-Hsp31p antibody (see the “Materials and methods” section for details). The results of this experiment, shown in Fig. 4, revealed that native Hsp31p migrated slightly faster under oxidative rather than under reductive conditions, similar to rxYFP, whereas Hsp31p lacking cysteine migrated at the same speed as the reduced native Hsp31p regardless of the redox environment. Hsp31p exhibited a smaller change in migration speed than rxYFP, which is likely due to the different number of cysteine residues (one versus two, respectively), while both proteins have similar molecular masses. Clearly, Cys138 in the Hsp31p polypeptide was oxidized under oxidative-stress conditions. Thus, its presence (and hence, its susceptibility to oxidative modification) was not a prerequisite for Hsp31p particle formation.

**Fig. 5:**
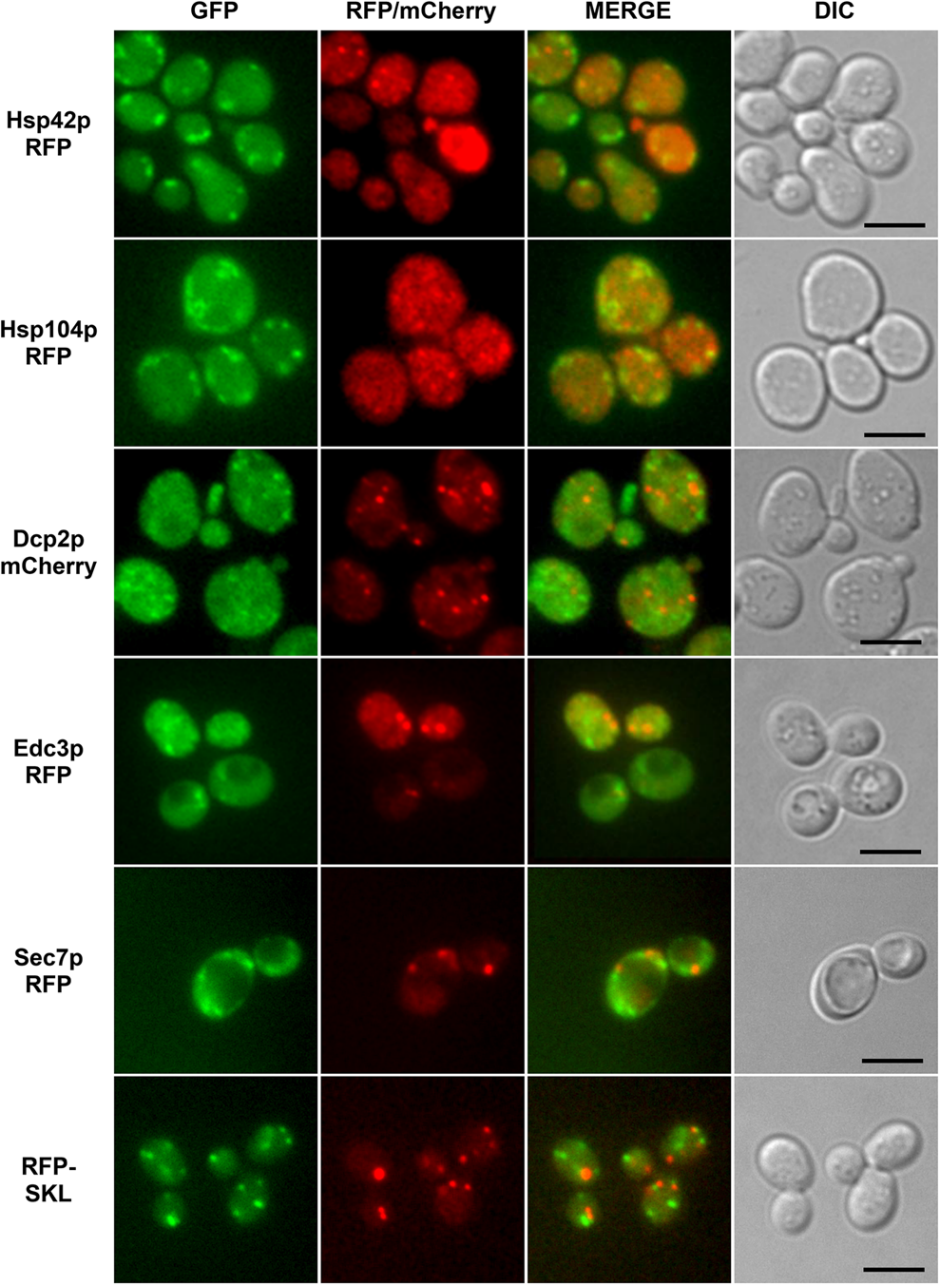
GFP-tagged Hsp31p does not colocalize with marker proteins of particulate structures found in budding yeast cells exposed to oxidative stress. Wild-type BY4741 cells were transformed with a single-copy YCp50-derivative plasmid expressing C-terminally GFP-tagged Hsp31p and with plasmids expressing markers of stress granules (Dcp2p-mCherry), P-bodies (Edc3p-RFP), Golgi apparatus (Sec7p-RFP) or peroxisomes (mRFP-SKL). Additionally, the colocalization of Hsp31p-GFP and Hsp42p-RFP or Hsp104p-RFP, all of which form particles in cells exposed to oxidative stress, was analyzed in the same manner. See Table 2 for the list of plasmids used in this experiment. The transformant strains were grown to mid-exponential phase in SC medium and then exposed to 0.1 mM cumene-OOH for 2 h. GFP, RFP, and mCherry fluorescence was examined on an Axio Imager.M2 fluorescence microscope using a 100× objective and 38HE-GFP or 20HE-rhodamine filter sets (Zeiss)

The formation of Hsp31p-GFP particles under some of the environmental conditions documented herein seems to agree with the findings of Miller-Fleming et al. (2014), who demonstrated the particulate distribution of Hsp31p-GFP under glucose-starvation and extreme heat stress (46 °C); however, these authors also noted Hsp31p-GFP particles within non-stressed and glucose-supplemented cells, which we did not observe here. Because these authors also examined the interaction of Hsp31p-GFP with components of P-bodies and stress granules in cells undergoing the diauxic shift, as well as its colocalization with P-bodies and stress granules under extreme heat stress (46 °C), we explored the possibility that the same structures contained Hsp31p-GFP under our stress conditions. However, as documented in Fig. 5, particulate Hsp31p-GFP under oxidative stress did not colocalize with P-bodies or stress granules. Moreover, particulate Hsp31p-GFP did not colocalize with other subcellular structures, such as the Golgi apparatus or peroxisomes, which might adopt a morphology similar to the observed Hsp31p-GFP granules.

### The abundance of Hsp31p-GFP foci under oxidative stress is influenced by the Ssa2p and Hsp104p chaperones

**Fig. 6:**
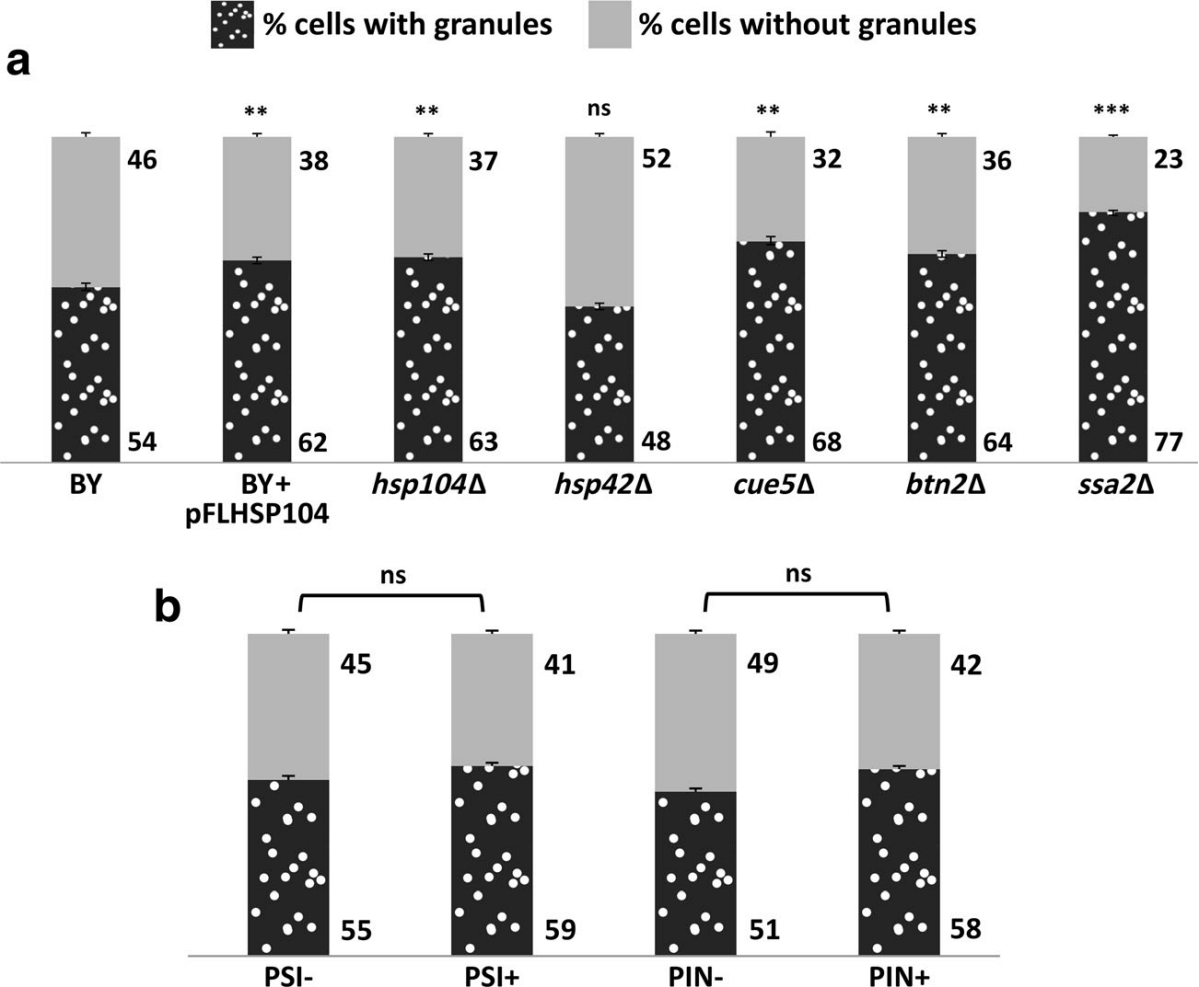
The assembly of Hsp31p-GFP particles is inhibited by cellular chaperone proteins but is not influenced by prion formation. (**a**) Wild-type strain (BY), the same strain overproducing the Hsp104p chaperone (BY + pFLHSP104) and the indicated deletion strains (*hsp104Δ*, *hsp42Δ*, *cue5Δ*, *btn2Δ*, and *ssa2Δ*) or (**b**) the strains containing the [PSI+] or [PIN+] prions together with their prionless isogenic parental strains (see Table 1 for the detailed strain descriptions) were transformed with a single-copy YCp-derivative plasmid expressing C-terminally tagged Hsp31p. The transformant strains were grown to mid-exponential phase in SC medium, exposed to 0.1 mM cumene-OOH for 2 h, visualized by fluorescence microscopy and scored for the presence of green fluorescence particles as described in Fig. 3 legend. Each data point is the average of three biological replicates, with 200 cells counted per replicate; the standard deviations are represented by error bars. Statistical significance was calculated with Student’s *t* test: *** *p* < 0.001, ** 0.001 < *p* < 0.05, ns *p* > 0.05. Significance in panel (**a**) refers to the first (BY) data point

To further investigate the nature of these particles, we searched for proteins that interacted physically with Hsp31p under stress conditions using metal-affinity chromatography and His-tagged Hsp31p as bait. We employed two stress conditions: oxidative stress and ethanol stress. No significant interactions with other proteins were found under oxidative stress; however, under ethanol stress, Ssa2p was the most prominent protein specifically retained on the His-Hsp31p-preloaded metal affinity resin (data not shown). This protein belongs to the HSP70 family of ATP-binding proteins, which are involved in protein folding and degradation (Bukau and Horwich 1998), vacuolar protein import (Satyanarayana et al. 2000), and yeast prion biogenesis (Jones and Tuite 2005). Therefore, Hsp31p-GFP particles might be protein aggregates, and Ssa2p might associate with them in performing its function. We examined this possibility by evaluating the frequency of Hsp31p-GFP particle formation in wild-type and isogenic *ssa2Δ* deletion strain. Indeed, Hsp31p-GFP particles were significantly more abundant in the absence of Ssa2p (see Fig. 6a). Thus, we next assessed the involvement of other chaperone proteins in the formation of Hsp31p-GFP foci. As shown in Fig. 6a, the absence of Hsp104p, another protein involved in the disassembly of aggregates, had similar consequences. The same phenomenon was observed when Hsp104p was overexpressed. Conversely, the absence of another chaperone protein (Hsp42p) had no effect on the formation of Hsp31p-GFP foci. Interestingly, Fig. 5 shows that under oxidative stress, both Hsp104p-RFP and Hsp42p-RFP formed or were associated with particles that appeared to be similar to Hsp31p-GFP granules. However, merging of the images demonstrated that these particles were separate entities.

To identify additional interactions between Hsp31p and other proteins, we screened publicly available images demonstrating the subcellular localization of most *S. cerevisiae* proteins fused to GFP (Tkach et al. 2012). We selected a dozen proteins with a particulate localization pattern resembling Hsp31p-GFP. We reasoned that if Hsp31p-GFP formed particles because it associated with particles formed by any of these proteins, then the absence of the respective gene would reduce or eliminate formation of the fluorescent particles. The respective deletion clones lacking genes encoding these proteins were transformed with the single-copy plasmid expressing Hsp31p-GFP, and their tendencies to form fluorescent particles under oxidative stress were compared with that of the wild-type strain. However, none of these clones displayed a diminished number of Hsp31p-GFP particles. Figure 6a shows the effects of the absence of two example proteins (Cue5p and Btn2p) selected for this experiment. Cue5p is a cytosolic ubiquitin-binding protein, whereas Btn2p contributes to prion curing and therefore may also be associated with protein disaggregation. Both proteins, together with Hsp42p, appeared in the context of aggregate formation and the biogenesis of IPOD, INQ and CytoQ, which are intercellular structures that sequester misfolded proteins (Miller et al. 2015). Clearly, the absence of Cue5p or Btn2p did not prevent the formation but instead slightly increased the number of Hsp31p-GFP foci.

### Hsp31p interactions with potential prion-forming proteins

The chaperone function of Hsp104p is known to influence the stability and abundance of prion particles (Jones and Tuite 2005). According to our observations, this function apparently affected the formation of Hsp31p-containing particles in a similar manner. Moreover, recent reports have indicated the involvement of Hsp31p in the maintenance of Sup35p-derived [PSI+] prions (Aslam et al. 2016). Thus, testing the mutual interdependence between the Hsp31p-GFP intracellular distribution under oxidative stress conditions and the presence of prion particles inside of cells seemed to be a reasonable approach. Assuming that fluorescent particles might form when Hsp31p-GFP binds to prions to fulfill its role as a chaperone, we examined whether the abundance of these particles was altered in the absence of genes encoding known prion-forming proteins. We examined the *cyc8Δ*, *mot3Δ*, *new1Δ*, *nup100Δ*, *pin2Δ*, *rnq1Δ*, *sfp1Δ*, *std1Δ*, and *ure2Δ* strains, lacking genes encoding proteins that form or induce the formation of the [OCT+], [MOT3+], [NU+], [NUP100+], [PIN+], [ISP+], [GAR+], and [URE3] prions, respectively. None of these deletion strains differed significantly from the wild-type parental strain with respect to the intracellular distribution of Hsp31p-GFP under oxidative stress conditions (data not shown). Therefore, none of the respective proteins appeared to be involved in the formation of Hsp31p-GFP-containing particles. In a complementary approach, we tested whether the presence of [PSI+] or [PIN+] prions in cells influenced the frequency of Hsp31p-GFP particle formation. As shown in Fig. 6b, the results of this experiment did not reveal any significant differences in this respect between the [PSI+] and [PSI−] or [PIN+] and [PIN−] cells.

## Discussion

The stress response, which pertains to all living organisms, is a vital element of their interactions with the environment. Despite being extensively studied, the stress response has not been fully elucidated. The number of genes that are crucial for stress survival is constantly growing and encompasses some genes that are seemingly unrelated to stress or of unknown function. We can expect even more genes to be revealed as stress responsive. *S. cerevisiae HSP31* is a relatively recent addition to the list of stress-responsive genes. For years, this gene has received little interest except as a human DJ-1 orthologue, but it has recently been extensively studied in *S. cerevisiae* (Natkańska et al. 2017; Aslam et al. 2016; Aslam and Hazbun 2016; Bankapalli et al. 2015; Amm et al. 2015; Tsai et al. 2015; Wilson 2014; Miller-Fleming et al. 2014). *HSP31* orthologues from related yeast species have also been investigated (Zhao et al. 2014; Hasim et al. 2014). We have previously demonstrated that the *HSP31* gene promoter responds to multiple environmental stresses, especially to fermentation products. We have also demonstrated that in its absence, cells become oversensitive to these stressors, and that Hsp31p possesses glyoxalase III activity in vivo. Our results suggest that this protein may have a broader range of substrates that remain to be identified (Natkańska et al. 2017). In the present article, we further assessed the role of Hsp31p in budding yeast cells by examining its intracellular localization under standard growth conditions and after exposure of the cells to various environmental stresses. To achieve this goal, we employed fusions of the full-length *HSP31* gene with the sequence encoding GFP. In the initial experiments, we used the strain bearing the chromosomal C-terminal *HSP31-GFP* fusion constructed as part of the whole-genomic protein localization study (Huh et al. 2003). Further experiments were conducted with constructs generated by our group (see Table 2). To test the potential influence of GFP tag placement, we prepared both C- and N-terminal fusions. Because presence of the conserved Cys138 residue in the Hsp31p polypeptide is necessary for its glyoxalase III activity, we also generated constructs encoding proteins lacking this residue. To ensure that the level of Hsp31p-GFP encoded by our constructs resembled that of native Hsp31p, expression of these gene fusions was controlled by the native *HSP31* promoter (upstream to −453 bp), and the constructs were introduced into yeast cells on the single-copy YCp50 vector.

Our results revealed that under standard growth conditions and after exposure to most of the tested stressors, Hsp31p-GFP was uniformly distributed in the cytosol regardless of the construct origin, GFP tag placement or presence of the Cys138 residue. However, Hsp31p-GFP became localized to particles in cells exposed to oxidative stress following treatment with 0.1 mM cumene-OOH or 0.2 mM tert-butyl-OOH or in post-diauxic cells (see Figs. 1, 2). Superficially, this particulate localization did not seem to depend on the tag placement or the presence of Cys138. However, when the fluorescent images were quantified, the C-terminal GFP fusion and the protein lacking Cys138 seemed to have a stronger tendency to form particles (see Fig. 3a, b). A much higher particle abundance was observed in the *yap1Δ* deletion strain, which was unable to respond to and protect itself from oxidative stress (see Fig. 3c). Although oxidative stress appeared to be elevated in these strains (Natkańska et al. 2017), the addition of an exogenous reactive oxygen species (ROS) generator was necessary to induce particulate Hsp31p-GFP localization. In the absence of this stressor, the protein was distributed uniformly in the cytosol of the *yap1Δ* strain (data not shown). The much higher number of Hsp31p-GFP particles in the *yap1Δ* strain suggests the synergy between oxidative stress and absence of the Yap1p transcription factor. Indeed, in *yap1Δ* cells, the levels of agents that dissipate ROS are low. Consequently, ROS concentrations will be much higher in *yap1Δ* cells than in wild-type cells following exposure to the same concentration of ROS generator. This suggests that the formation of Hsp31p-GFP particles is a direct consequence of oxidative stress, perhaps the manifestation of its damaging power.

Notably, the particles were not observed in ethanol- or methylglyoxal-treated cells, even though these stressors induced stronger Hsp31p-GFP expression than cumene-OOH or tert-butyl-OOH (see Fig. 1 and Natkańska et al. 2017). Ethanol and methylglyoxal were postulated to induce oxidative stress, and ROS were considered mediators of cellular responses to these stressors. Apparently, endogenous ROS accumulation in *yap1Δ* cells or in cells treated with ethanol or methylglyoxal was not sufficient to induce the formation of Hsp31p-GFP particles. As documented in Fig. 4, oxidative stress caused the oxidation of Hsp31p Cys138; however, the formation of Hsp31p-GFP particles was independent of this reaction because Hsp31p-GFP lacking this residue aggregated even more frequently.

This type of change in subcellular localization is observed quite often. The whole-genomic and proteomic screens assessing alterations in the intracellular distribution of budding yeast proteins in response to altered cellular environments have identified many proteins that are cytosolic under standard growth conditions and become particulate following exposure to stress (Tkach et al. 2012; Breker et al. 2013) or starvation (Narayanaswamy et al. 2009). Curiously, Hsp31p was not mentioned in those studies.

Thus, the most immediate question concerned the identity of the observed particles. We attempted to answer this question using fluorescent markers of various cellular organelles or structures that might resemble the Hsp31p-GFP particles. We tested P-bodies, stress granules, the Golgi apparatus, and peroxisomes, but consistently found no signs of colocalization (see Fig. 5). A noticeable feature of Hsp31p-GFP particles revealed by fluorescence microscopy analysis was their variable morphologies. However, as seen in Figs. 1 and 2, particle morphology does not seem to be correlated with the specific stress conditions used to stimulate Hsp31p-GFP aggregation. This same variability is illustrated by the example images of cumene-OOH-induced Hsp31p-GFP particles shown in Fig. 5. However, regardless of particle appearance in the individual cells, colocalization with organellar markers was not observed. The same was true for tert-butyl-OOH-induced Hsp31p-GFP particles (data not shown).

To further explore the intracellular localization of Hsp31p and to determine its associated structures, we attempted to identify proteins that physically interacted with Hsp31p under stress conditions. We expected to identify Hsp31p-interacting proteins known to be localized to certain cellular particulate structures. Another goal of this study was based on recent reports postulating a chaperone function for Hsp31p (Aslam et al. 2016; Tsai et al. 2015). The ability of Hsp31p to prevent aggregation was demonstrated both in vitro and in vivo with various heterologous substrates. If Hsp31p also exhibited chaperone activity toward native budding yeast proteins, we would expect to find these proteins among the physically interacting proteins. In this experiment, we used metal affinity chromatography with His-tagged Hsp31p as bait. The only protein that was specifically retained on the Hsp31p-His_6_-preloaded metal affinity resin was Ssa2p, which was itself an abundant chaperone protein. Upon further exploration, we found that in the absence of Ssa2p or Hsp104p (another chaperone protein involved in preventing cellular proteins from aggregating), Hsp31p-GFP particles were significantly more abundant. Thus, we preliminarily concluded that these particles were more likely to be aggregates of Hsp31p-GFP alone and that their formation was partially mitigated by Ssa2p or Hsp104p rather than the aggregates of some other protein(s) to which Hsp31p-GFP binds to perform its postulated chaperone (or co-chaperone together with Ssa2p) functions (Aslam et al. 2016; Tsai et al. 2015). This conclusion discouraged us from further inquiries into a potential functional relationship between Hsp31p and Ssa2p. Notably, Hsp104p was also found in particles under cumene-OOH-induced oxidative stress, but it did not colocalize with Hsp31p-GFP (see Fig. 5).

Oxidative stress conditions increase the tendency of intracellular proteins to aggregate (Weids et al. 2016). These conditions also induce de novo prion particle formation (Doronina et al. 2015). Therefore, we considered another explanation for the appearance of Hsp31p-GFP particles: binding to certain categories of proteins that are misfolded following oxidative stress. We tested the possibility that Hsp31p-GFP associates with prion proteins. We did not observe any significant influence of the absence of known proteins with potential prion-forming abilities in budding yeast cells on Hsp31p-GFP particle abundance (data not shown). Additionally, the number of Hsp31p-GFP particles did not differ between the strains containing or lacking the [PIN+] or [PSI+] prions (see Fig. 6b). As shown in Fig. 6a, both deletion and overexpression of the *HSP104* gene increased the frequency of Hsp31p-GFP particle formation. These differences, although minor, were significant. This correlation resembles the dependence of prion formation and propagation on the Hsp104p chaperone. Moreover, prion formation is greatly enhanced under oxidative stress (Doronina et al. 2015), and the same is true for Hsp31p-GFP particle formation. Nevertheless, we consider these observations insufficient to claim that Hsp31p is capable of prion formation, although as recently shown, the number of proteins that potentially form prion particles is much larger than the number of prion proteins identified to date (Chakrabortee et al. 2016).

Notably, Hsp31p-GFP particle formation is not dependent on the functionality of this protein (i.e., the presence and oxidation state of Cys138). Moreover, these particles are completely absent when Hsp31p synthesis is strongly induced, i.e., in cells exposed to a high ethanol concentration or to methylglyoxal, which is a substrate for Hsp31p enzymatic activity. This finding suggests that the formation of particles has little if anything to do with the intracellular function of Hsp31p.

Particulate Hsp31p-GFP in *S. cerevisiae* cells exposed to glucose deprivation or to heat stress has been previously shown by Fleming et al., and its colocalization with stress granules and P-bodies has been demonstrated (Miller-Fleming et al. 2014). Thus, our observations appear to be very consistent with these data, and they expand our knowledge of Hsp31p-GFP particle formation. However, we excluded the colocalization of Hsp31p-GFP with P-bodies and other particulate structures within yeast cells. Thus, the nature of the oxidative stress-induced Hsp31p-GFP particles described by our group seems to differ from the particles described by Fleming et al.

Taken together, our data strongly suggest that the appearance of particles is not linked to any physiological function. Instead, our results support the hypothesis that Hsp31p-GFP simply aggregates into particles under oxidative stress conditions. Our data indicate that the functional protein is uniformly distributed in the cytosol. Further studies are necessary to reveal the functional implications of this localization.
